# T and B cell responses following primary COVID-19 vaccination with CoronaVac and two heterologous BNT162b2 booster doses

**DOI:** 10.1128/msphere.00722-25

**Published:** 2025-11-18

**Authors:** Apirath Wangteeraprasert, Sutatip Pongcharoen, Jatuporn Ngoenkam, Supawadee Makanut

**Affiliations:** 1 Division of Immunology, Department of Internal Medicine, Faculty of Medicine, Naresuan University 59212, Phitsanulok, Thailand; 2 Department of Microbiology and Parasitology, Faculty of Medical Science, Naresuan University 59212, Phitsanulok, Thailand; 3 Division of Pulmonology, Department of Internal Medicine, Faculty of Medicine, Naresuan University 59212, Phitsanulok, Thailand; Instituto de Biotecnologia/UNAM, Cuernavaca, Morelos, Mexico

**Keywords:** memory T cell, memory B cell, COVID-19 vaccines, CD4+ T cell, CD8+ T cell

## Abstract

The present study investigated T and B cell responses following a second heterologous booster dose of BNT162b2 administered after a two-dose CoronaVac regimen for coronavirus disease 2019 (COVID-19) vaccination in 15 healthcare workers. Blood samples were collected 4 weeks after the first booster and at both 4 and 24 weeks after the second BNT162b2 booster. Interferon-γ-secreting CD4+ and CD8+ T cells were detectable 4 weeks after the first booster, whereas only CD4+ T cells remained detectable at both 4 and 24 weeks after the second booster. Seven of the 15 participants (46.7%) were diagnosed with COVID-19 approximately 16 weeks after receiving the second booster. These individuals exhibited significantly higher frequencies of CD4+ T cells at 24 weeks post-booster than at 4 weeks post-booster. In contrast, the non-COVID-19 group exhibited significantly higher CD4+ T cell responses 4 weeks after the second booster. Memory B cells were detected at low frequencies at all three time points. IgG antibodies against the severe acute respiratory syndrome coronavirus 2 (SARS-CoV-2) spike protein were detectable at all three time points, with a significant decline observed 24 weeks after the second booster. Overall, CD4+ T and B cell responses induced by a heterologous second booster dose of BNT162b2 following a primary two-dose CoronaVac regimen were rapidly elicited and sustained for at least 6 months.

**IMPORTANCE:**

There is limited evidence regarding T and B cell responses following a primary COVID-19 vaccination series with CoronaVac and two heterologous BNT162b2 booster doses. This study investigated the longitudinal T and B cell responses induced by a second heterologous BNT162b2 booster following a primary two-dose CoronaVac COVID-19 vaccination regimen. These results demonstrate that CD4+ T cells induced by the second heterologous BNT162b2 booster play a key role in protection against SARS-CoV-2 infection and progression to severe disease. This study suggests the need for the future consideration of repeated emergency vaccine-boosting strategies in response to emerging viral infections.

## INTRODUCTION

The coronavirus disease 2019 (COVID-19) outbreak in late 2019 infected over 700 million individuals and resulted in more than 7 million deaths worldwide (1). Severe acute respiratory syndrome coronavirus 2 (SARS-CoV-2), the causative agent of COVID-19, has been studied to elucidate its infection mechanisms and host immune responses with the aim of developing effective prevention strategies. Various types of COVID-19 vaccines, including inactivated viruses, viral vectors, and mRNA vaccines, have been developed by vaccine manufacturers and approved for emergency use by the World Health Organization (WHO) (2). Inactivated vaccines, which are a traditional and well-established platform, contain whole SARS-CoV-2 particles that have been chemically killed, thereby inducing an immune response without causing disease. CoronaVac is produced via β-propiolactone inactivation, which damages viral spike proteins and prevents their binding to host cells (3). This approach predominantly elicits antibody-mediated responses targeting multiple viral components, because there are no infected cells. Additionally, inactivated vaccines require booster doses because of their low production titers (4). An mRNA vaccine, namely BNT162b2, was the first COVID-19 vaccine approved by the United States in August 2021. This is a lipid nanoparticle-encapsulated nucleoside-modified RNA vaccine that encodes a pre-fusion-stabilized, membrane-anchored, full-length SARS-CoV-2 spike (S) protein. Once delivered into host cells, the mRNA is translated into S protein, which in turn activates robust immune responses. However, this vaccine requires stringent storage and handling conditions because of its inherent instability (5, 6). The most recently developed platform is the protein subunit vaccine. NVX-CoV2373 is a recombinant protein vaccine consisting of SARS-CoV-2 spike trimer nanoparticles formulated with the saponin-based Matrix-M adjuvant. A major advantage of this approach is that it delivers only the essential antigens necessary to stimulate immunity, often resulting in fewer adverse events than with other vaccine types (7).

In April 2021, during the COVID-19 pandemic, the Ministry of Public Health of Thailand launched the first COVID-19 vaccination campaign for healthcare workers, in which two doses of an inactivated virus vaccine, namely CoronaVac, were administered 4 weeks apart. This vaccine demonstrated an overall efficacy of approximately 50.7%–83.5% in preventing symptomatic COVID-19 infection 14 days or more after the second dose (8–11). However, in August 2021, the SARS-CoV-2 Delta variant (B.1.617.2), characterized by mutations in the spike protein gene, was identified as having increased transmissibility and rapidly spreading worldwide (12). In December 2021, the Omicron variant (B.1.1.529) was also reported. The Thai Ministry of Public Health administered the first and second booster doses of the mRNA COVID-19 vaccine (BNT162b2) to healthcare workers in August 2021 and between January and February 2022, respectively. This vaccine had an efficacy of 95% in preventing COVID-19 at 7 days after the second dose (13). Current evidence indicates that heterologous booster vaccination elicits a robust immune response and offers enhanced protection (14, 15).

T cells initiate an early immune response within 2 weeks of SARS-CoV-2 infection (16). Both CD4+ and CD8+ T cell responses can be detected in the early period following the first vaccination and play a critical role in protection against COVID-19 infection (17, 18). Additionally, COVID-19 vaccines stimulate B cells to produce neutralizing antibodies.

Few studies have investigated T and B cell responses following heterologous booster vaccination (19–22). Nevertheless, knowledge of T and B cell responses following a heterologous second booster dose of BNT162b2 after a two-dose CoronaVac vaccination is limited. Therefore, the present study aimed to evaluate T and B cell responses induced by a heterologous second booster dose of BNT162b2 following a primary two-dose CoronaVac COVID-19 vaccination regimen in Thai healthcare workers.

## MATERIALS AND METHODS

### Study design and participants

**Fig 1:**
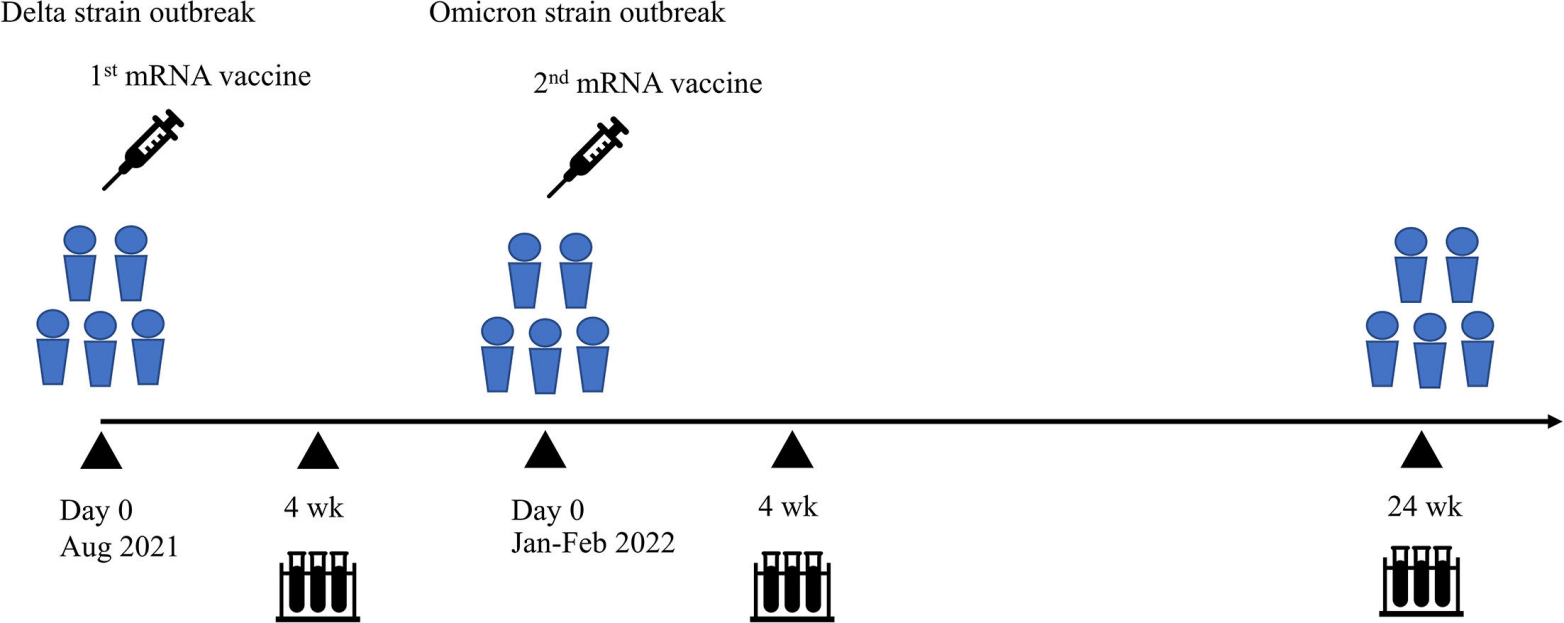
The first COVID-19 vaccination campaign in Thailand began in April 2021 with two doses of inactivated COVID-19 vaccine (CoronaVac). In August 2021, following the spread of the Delta variant, a heterologous mRNA vaccine (BNT162b2) was offered a first booster dose to healthcare workers who had completed the two-dose CoronaVac regimen. However, the delta wave was soon followed by the emergence of the Omicron variant in December 2021. A second BNT162b2 booster dose was administered in January and February 2022. Blood samples were collected at 4 weeks after the first booster and at 4 and 24 weeks after the second booster dose of BNT162b2.

A longitudinal prospective descriptive study was conducted by recruiting 20 adults (10 men and 10 women) among healthcare workers in the Faculty of Medicine, Naresuan University, Thailand, who received two doses of CoronaVac followed by two heterologous booster doses of BNT162b2 for COVID-19 vaccination between April 2021 and February 2022 and agreed to donate 20 mL of blood. All vaccine doses were provided by the Thai Ministry of Public Health. Eligible adult participants were aged 20 years or older with no prior clinical symptoms of SARS-CoV-2 infection, no history of close contact with COVID-19 patients, and no previous diagnosis of SARS-CoV-2 infection. The exclusion criteria were immunocompromised individuals, those receiving immunosuppressive therapy or blood products, pregnant women, individuals with other severe infections, and those diagnosed with COVID-19 within 14 days of blood sampling. Clinical data, including age, sex, and COVID-19 status, were collected during all visits. Blood samples were obtained 4 weeks after the first booster dose, and at 4 and 24 weeks after the second booster dose of BNT162b2 (Fig. 1).

### Cell preparation

Venous blood samples were collected in tubes containing ethylenediaminetetraacetic acid. Peripheral blood mononuclear cells (PBMCs) were isolated by standard density gradient centrifugation with Lymphoprep (Serumwerk, Bernberg, Germany). PBMCs were used to study SARS-CoV-2-specific B cells. CD4+ T cells were isolated by depleting CD8+ T cells using Human CD8+ T cell-positive selection kits (Miltenyi Biotec, CA, USA). For the analysis of CD8+ T cells, CD4+ T cells were depleted using Human CD4+ T cell isolation kits (Miltenyi Biotec). The purity of the isolated T cell population (>90%) was confirmed by flow cytometry.

### Interferon-γ ELISpot assay

Interferon-γ (IFN-γ) secretion from T cells in response to SARS-CoV-2 peptides was measured using an IFN-γ ELISpot assay kit (catalog no. 3420-2APT-10, batch 404; Mabtech, AB, Sweden) according to the manufacturer’s instructions. The precoated 96-well plate was washed four times with 200 µL of sterile phosphate-buffered saline (PBS), followed by blocking with 200 µL AIM-V medium (Thermo Fisher Scientific, MA, USA) for 30 min at room temperature. The medium was replaced with 100 µL of either anti-CD3 antibody (provided in the kit) at a final concentration of 1 µg/mL as a positive control; dimethyl sulfoxide (Sigma-Aldrich, MA, USA) as a negative control; or with 2 µg/mL of individual peptides, including the spike protein (S), S1 domain, and NMO peptide pools (all from Mabtech, Sweden). NMO peptides consist of nucleoproteins (N), membrane proteins (M), and open reading frames.

A total of 200,000 CD4+ or CD8+ T cells in 100 µL of medium were added to each well and incubated for 20 h in a humidified incubator at 37°C with 5% CO_2_. After five washes with PBS, 100 µL of PBS-diluted 7-B6-ALP detection antibody (provided in the kit) was added to each well and incubated for 2 h. The plate was then washed again five times with PBS before adding 100 µL of ready-to-use BCIP/NBT-plus substrate (provided in the kit), followed by a brief incubation in the dark for 2 min. The supernatant was discarded, and the plate was washed five times with PBS to stop the reaction. The plate was then air-dried, and spots were counted using an ELISpot reader (CTL, Cellular Technologies Ltd., USA). Detected spots represented IFN-γ-producing cells. Spots from untreated wells were used to normalize the results of the positive and test conditions. The data are presented as the number of specific spot-forming cells (SFCs) per 1 × 10^6^ PBMCs. Positive responses were defined as a mean spot-forming unit (SFU) count ≥30 above the negative control wells (23).

### Estimation of RBD-specific memory B cells

PBMCs (2 × 10^6^) were cultured in complete RPMI medium supplemented with 10% fetal bovine serum (FBS), 1 µg/mL R848 (TLR7/8 agonist), and 10 ng/mL recombinant human IL-2 (Thermo Fisher), or left unstimulated. Cells were incubated for 72 h at 37°C in a 5% CO_2_ atmosphere, as previously reported (24). Prior to cell seeding, ELISpot plates (Mabtech, Mountain View, CA, USA) were washed four times with PBS and blocked with complete medium for 30 min at room temperature. After 72 h of stimulation, the cells were harvested and washed twice with complete medium by centrifugation at 1,500 rpm for 10 min. The cell concentration was adjusted to 5 × 10^6^ cells/mL.

A total of 5 × 10^5^ PBMCs in 100 µL of complete medium were added to each well, pre-assigned as either negative control or test conditions. To assess total IgG-secreted memory B cells, 10 µL of treated PBMCs was seeded into wells designated for total IgG detection. Complete medium was added to bring the final volume of 150 µL per well, followed by incubation for 18–24 h at 37°C in a 5% CO_2_ incubator. After incubation, the supernatants were discarded, and the plates were washed five times with PBS. For the detection of receptor-binding domain (RBD)-specific IgG, 100 µL of RBD-WASP (diluted in PBS, provided in the kit) was added to the corresponding test wells. For the detection of total IgG, 100 µL of 1 µg/mL MT78/145-biotin (in PBS + 0.5% FBS, kit-supplied) was added to total IgG wells. Negative control wells received 100 µL of PBS + 0.5% FBS. Following a 2 h incubation at room temperature, the plates were washed as described above. Subsequently, 100 µL of anti-WASP-ALP (diluted in PBS + 0.5% FBS) was added to RBD-specific IgG and negative control wells, while 100 µL of streptavidin-ALP (in PBS + 0.5% FBS) was added to total IgG wells. The plates were incubated for 1 h at room temperature and washed again. The spots were developed using 100 µL of BCIP/NBT-plus substrate with 2 min of incubation in the dark. The plates were washed with distilled water and air-dried. SFUs were quantified using an ELISpot reader (CTL, Cellular Technologies Ltd.). SFUs from untreated wells were used for background normalization. The results were expressed as the number of specific SFCs per 1 × 10^6^ PBMCs. A positive response was defined as a mean SFU ≥ 30 above the corresponding negative control.

### IgG antibody measurement

Venous blood samples (6 mL each) were collected into EDTA tubes. The samples were centrifuged at 1,500 rpm for 10 min to separate the serum, which was subsequently stored at −20°C until antibody analysis. IgG antibodies specific to the SARS-CoV-2 spike protein were quantified using a fluoroenzyme immunoassay (EliA SARS-CoV-2-Sp1 IgG; Thermo Fisher Scientific, Waltham, MA, USA). Antibody titers were interpreted as follows: negative (<7 U/mL), equivocal (7–10 U/mL), or positive (>10 U/mL). A titer exceeding 10 U/mL was considered indicative of a positive serological response against the SARS-CoV-2 spike protein. According to the Department of Medical Sciences, Ministry of Public Health, Thailand, this assay demonstrated a diagnostic sensitivity of 98% and specificity of 100% (analysis no. CV93). Previous studies have reported diagnostic sensitivities ranging from 96.9% to 100% and specificities between 99.4% and 100% (25–28).

### Statistical analysis

Categorical variables are presented as frequencies and percentages. The distribution of continuous variables was assessed using the Shapiro–Wilk test. Friedman’s test was used to compare more than two related groups. For paired data, the Wilcoxon matched-pairs signed-rank test was used to analyze the medians and interquartile ranges (IQR; Q1, Q3). Comparisons between two independent groups were performed using the Mann–Whitney *U* test. Statistical significance was set at *P* < 0.05. All statistical analyses were conducted using the STATA software (version 17.0; StataCorp, College Station, TX, USA).

## RESULTS

### Participants

We recruited 10 men and 10 women who received two doses of CoronaVac followed by two booster doses of BNT162b2 for COVID-19 vaccination. Overall, 15 participants completed the follow-up periods. The mean age was 35.40 ± 5.22 years. The mean age of men and women was 33.14 ± 5.34 and 37.38 ± 4.53 years, respectively (Table 1).

**TABLE 1:** Demographic data of the participants

Parameter	Overall	Male	Female
Gender, *n* (%)	15	7 (46.67)	8 (53.33)
Age, mean ± SD (min, max)	35.40 ± 5.22 (26, 45)	33.14 ± 5.34 (26, 41)	37.38 ± 4.53 (30, 45)

### T cell response after vaccination

CD4+ and CD8+ T cell responses were measured 4 weeks after the first booster dose and at 4 and 24 weeks after the second booster dose of BNT162b2 using ELISpot assays. However, 7 out of 15 (46.7%) participants developed mild upper respiratory tract symptoms and were subsequently confirmed to have COVID-19 by rapid antigen testing approximately 16 weeks after the second booster. Therefore, CD4+ and CD8+ T cell responses to SARS-CoV-2 antigens were compared between participants with and without COVID-19.

First, we compared the CD4+ and CD8+ T cell responses. Four weeks after the first booster dose of BNT162b2, both CD4+ and CD8+ T cells responded to COVID-19 antigens (S, S1, and NMO) by secreting IFN-γ. Although IFN-γ-secreting CD4+ T cells were detected in slightly higher numbers than CD8+ T cells, the difference was not statistically significant. At 4 and 24 weeks after the second booster, CD4+ T cells continued to respond to the COVID-19 antigens (S, S1, and NMO), whereas CD8+ T cell responses were no longer detectable (Table 2).

**TABLE 2:** Comparison of CD4+ and CD8+ T cell responses at 4 weeks after the first booster and at 4 and 24 weeks after the second BNT162b2 booster

Parameter	CD4+ T cells, median (Q1, Q3), *n* = 15	CD8+ T cells , median (Q1, Q3), *n* = 15	*P* value^*a*^
4 weeks after the first BNT162b2 booster			
S	90 (45, 130)	40 (0, 80)	0.140
S1	105 (55, 180)	80 (0, 145)	0.443
NMO	80 (55, 210)	40 (5, 90)	0.268
4 weeks after the second BNT162b2 booster			
S	20 (0, 40)	0 (0, 20)	0.084
S1	45 (0, 80)	0 (0, 30)	**0.008^*b*^**
NMO	5 (0, 30)	0 (0, 15)	0.595
24 weeks after the second BNT162b2 booster			
S	60 (40, 140)	0 (0, 15)	**0.005^*b*^**
S1	100 (45, 195)	0 (0, 20)	**<0.001^*b*^**
NMO	50 (30, 85)	0 (0, 0)	**<0.001^*b*^**

^*a*^ Wilcoxon matched-pairs signed-rank test.

^*b*^ *P* values <0.05 were considered significant and are shown in bold.

Subsequently, CD4+ and CD8+ T cell responses were compared at 4 weeks after the first booster and at 4 and 24 weeks after the second booster doses of BNT162b2. CD4+ T cells were detected in significantly higher numbers in response to COVID-19 antigens (S, S1, and NMO) 4 weeks after the first booster than after the second booster. In contrast, CD8+ T cell responses were detectable at low levels 4 weeks after the first booster but were undetectable at 4 and 24 weeks after the second booster. A significant increase in CD4+ T cell responses to NMO antigens was observed at 24 weeks compared to that at 4 weeks after the second booster (Table 3).

**TABLE 3:** Comparison of CD4+ T cell, CD8+ T cell, B cell, and IgG antibody responses at 4 weeks after the first booster and at 4 and 24 weeks after the second BNT162b2 booster

Parameter	Group 1: 4 weeks after the first BNT162b2 booster, median (Q1, Q3), *n* = 15	Group 2: 4 weeks after the second BNT1612b2 booster, median (Q1, Q3), *n* = 15	Group 3: 24 weeks after the second BNT162b2 booster, median (Q1, Q3), *n* = 15	*P* value^*a*^	*P* value^*b*^, 1 vs 2	*P* value^*b*^, 1 vs 3	*P* value^*b*^, 2 vs 3
CD4+ T cells							
S	90 (45, 130)	20 (0, 40)	60 (40, 140)	**0.032^*c*^**	**0.020^*c*^**	0.836	0.094
S1	105 (55, 180)	45 (0, 80)	100 (45, 195)	**0.049^*c*^**	**0.041^*c*^**	0.730	0.163
NMO	80 (55, 210)	5 (0, 30)	50 (30, 85)	**0.012^*c*^**	**0.002^*c*^**	0.413	**0.025^*c*^**
CD8+ T cells							
S	40 (0, 80)	0 (0, 20)	0 (0, 15)	**0.009^*c*^**	**0.008^*c*^**	**0.032^*c*^**	0.780
S1	80 (0, 145)	0 (0, 30)	0 (0, 20)	**0.008^*c*^**	**0.002^*c*^**	**0.021^*c*^**	0.651
NMO	40 (5, 90)	0 (0, 15)	0 (0, 0)	**0.001^*c*^**	**0.002^*c*^**	**0.002^*c*^**	0.400
B cells	126 (6, 303)	28 (12, 142)	40 (4, 84)	0.189	0.191	0.073	0.691
IgG antibody	458 (210, 703)	301 (183, 480)	56 (43, 349)	**0.003^*c*^**	**0.009^*c*^**	**0.007^*c*^**	0.088

^*a*^ Friedman's test.

^*b*^ Wilcoxon matched-pairs signed-rank test.

^*c*^ *P* values <0.05 were considered significant and are shown in bold.

**Fig 2:**
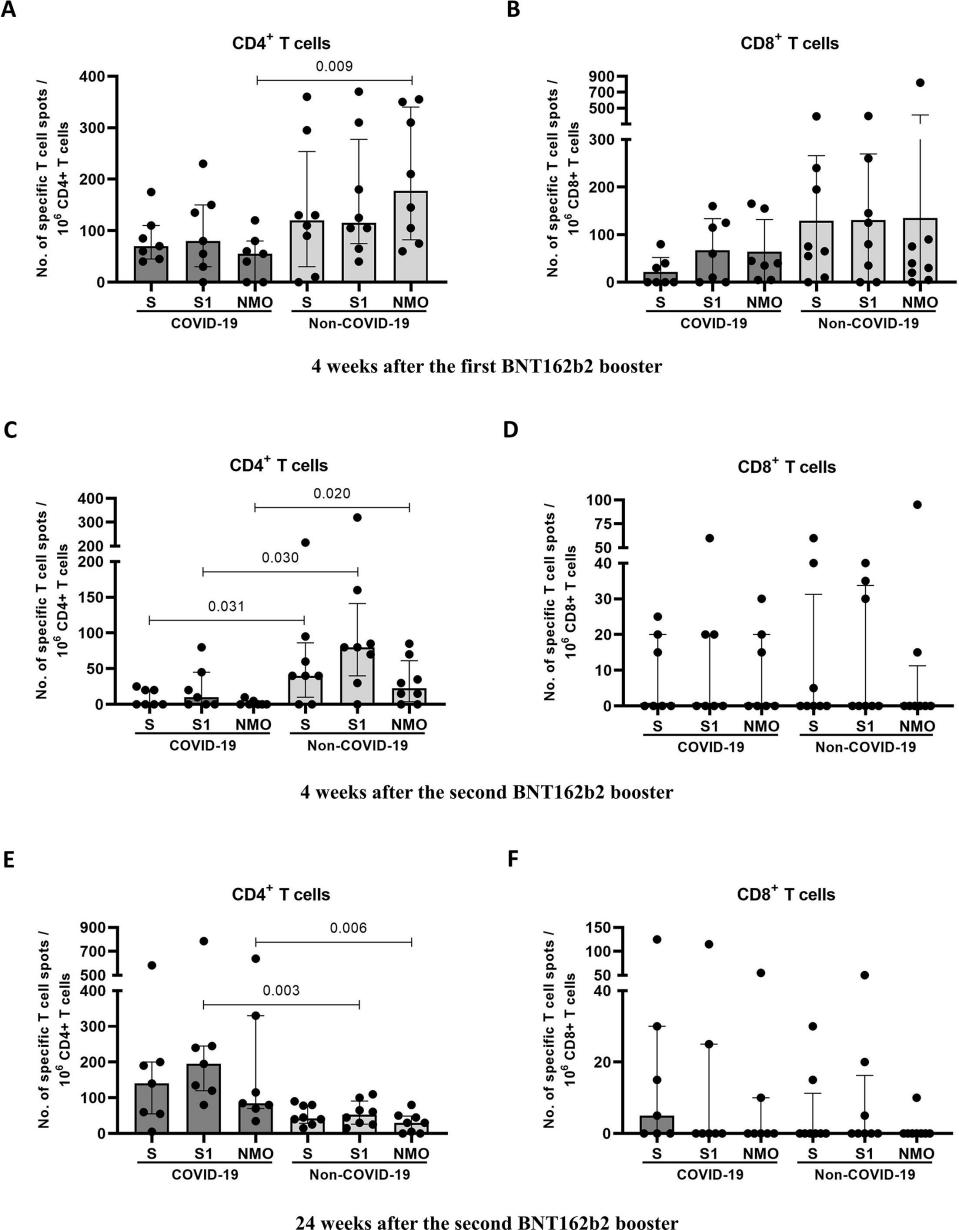
Subgroup analysis of CD4+ and CD8+ T cell responses in the COVID-19 and non-COVID-19 groups at 4 weeks after the first booster (**A, B**), 4 weeks (**C, D**), and 24 weeks (**E, F**) after the second BNT162b2 booster. *P* values <0.05 were considered statistically significant using the Mann–Whitney test.

Of the seven participants who contracted COVID-19 approximately 16 weeks after the second booster, none experienced severe disease, hospitalization, ICU admission, or death. Among the participants in the COVID-19 group, significantly higher CD4+ T cell responses to S, S1, and NMO antigens were observed at 24 weeks than at 4 weeks after the second booster. In contrast, no significant differences in CD4+ T cell responses were observed between the two time points in the non-COVID-19 group. CD8+ T cell responses were undetectable in both the COVID-19 and non-COVID-19 groups at both time points. A comparison between the COVID-19 and non-COVID-19 groups at the same time point revealed that the non-COVID-19 group exhibited significantly higher CD4+ T cell responses to the S, S1, and NMO antigens 4 weeks after the second booster. However, 24 weeks after the second booster, the COVID-19 group showed significantly higher CD4+ T cell responses to S1 and NMO antigens than the non-COVID-19 group (Table 4; Fig. 2C through F).

**TABLE 4:** Subgroup analysis of CD4+ T cell, CD8+ T cell, B cell, and IgG antibody responses in COVID-19 and non-COVID-19 participants at 4 and 24 weeks after the second BNT162b2 booster

Parameter	Comparison within COVID-19 at different time points			Comparison within non-COVID-19 at different time points			Comparison between COVID-19 and non-COVID-19 at the same time point	
	4 weeks, median (Q1, Q3), *n* = 7	24 weeks, median (Q1, Q3), *n* = 7	*P* value^*a*^	4 weeks, median (Q1, Q3), *n* = 8	24 weeks, median (Q1, Q3), *n* = 8	*P* value^*a*^	4 weeks: *P* value^*b*^	24 weeks: *P* value^*b*^
CD4+ T cells								
S	0 (0, 20)	140 (55, 200)	**0.028^*c*^**	40 (20, 77.5)	42.5 (32.5, 79)	0.726	**0.031^*c*^**	0.105
S1	10 (0, 45)	195 (120, 245)	**0.022^*c*^**	80 (50, 122.5)	53 (27.5, 82.5)	0.292	**0.030^*c*^**	**0.003^*c*^**
NMO	0 (0, 5)	85 (70, 330)	**0.018^*c*^**	22.5 (7.5, 52.5)	30 (2.5, 47.5)	0.944	**0.020^*c*^**	**0.006^*c*^**
CD8+ T cells								
S	0 (0, 20)	5 (0, 30)	0.246	0 (0, 22.5)	0 (0, 7.5)	0.085	1.000	0.239
S1	0 (0, 20)	0 (0, 25)	0.567	0 (0, 32.5)	0 (0, 12.5)	1.000	0.990	0.945
NMO	0 (0, 20)	0 (0, 10)	0.703	0 (0, 7.5)	0 (0, 0)	0.461	0.535	0.407
B cells	22 (12, 464)	58 (4, 102)	1.000	29 (13, 118)	40 (4, 66)	0.624	0.487	0.524
IgG antibody	408 (301, 624)	349 (234, 515)	1.000	244 (153.5, 309.5)	43 (34.5, 49.5)	**0.012^*c*^**	**0.043^*c*^**	**0.001^*c*^**

^*a*^ Wilcoxon matched-pairs signed-rank test.

^*b*^ Mann–Whitney test.

^*c*^ *P* values <0.05 were considered significant and are shown in bold.

### B cell response after vaccination

Low IgG-secreting memory B cell responses were detectable at 4 weeks after the first booster and at 4 and 24 weeks after the second booster dose of BNT162b2. The response at 4 weeks after the first booster was slightly higher than that at 4 and 24 weeks after the second booster; however, the difference was not statistically significant. Notably, IgG antibodies against the SARS-CoV-2 spike protein were detectable at all three time points, with significantly lower levels observed at 4 and 24 weeks after the second booster than at 4 weeks after the first booster (Table 3).

Subgroup analysis of the COVID-19 and non-COVID-19 groups revealed no significant differences in B cell responses between the two groups, with responses remaining low at both 4 and 24 weeks after the second booster. No significant differences in IgG levels were observed between weeks 4 and 24 after the second booster in the COVID-19 group. Conversely, IgG levels were significantly decreased at 24 weeks in the non-COVID-19 group.

**Fig 3:**
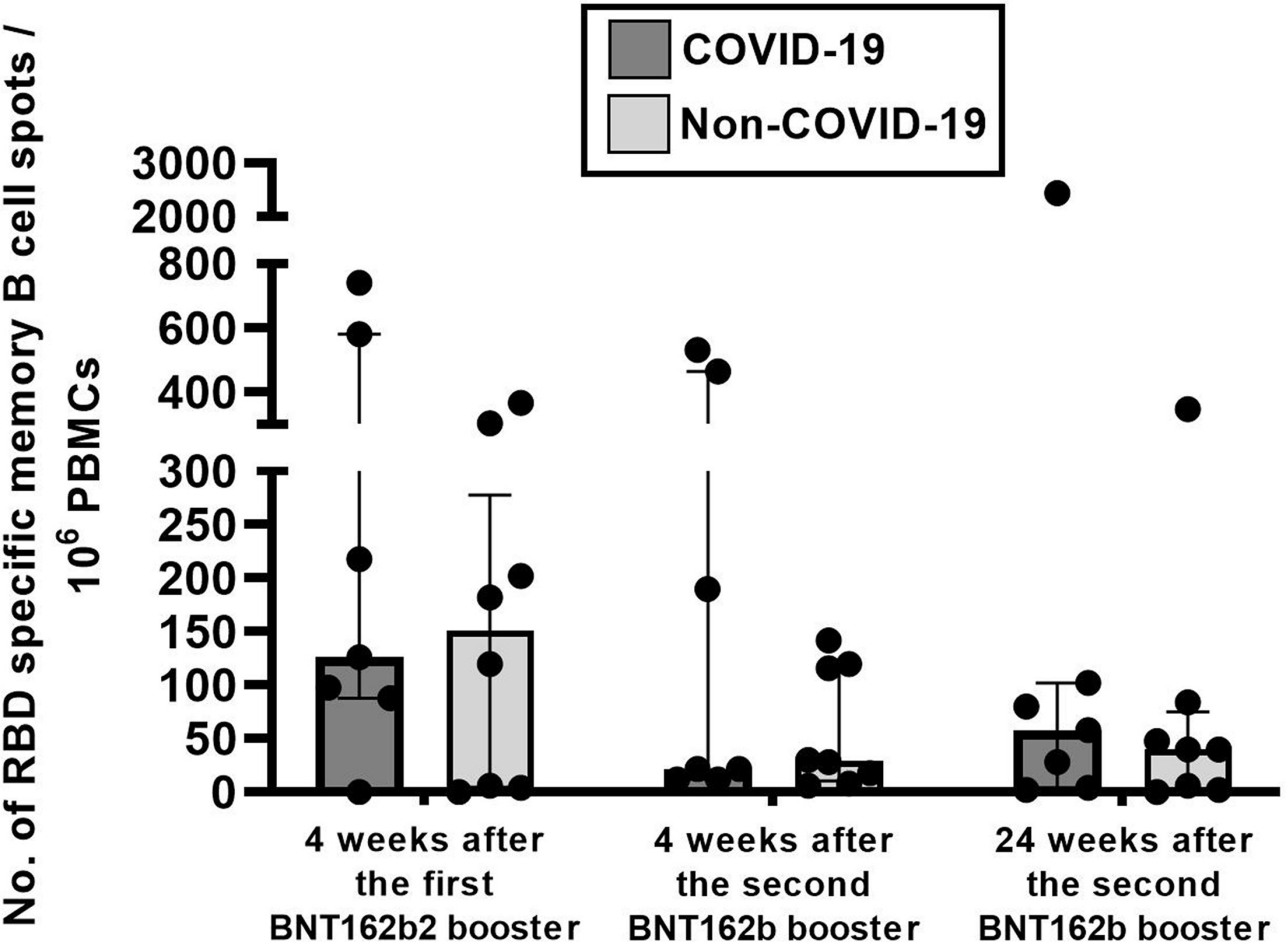
Subgroup analysis of B cell responses in the COVID-19 and non-COVID-19 groups at 4 weeks after the first booster, 4 weeks, and 24 weeks after the second BNT162b2 booster. *P* values <0.05 were considered statistically significant using the Mann–Whitney test.

A comparison between the COVID-19 and non-COVID-19 groups at the same time point revealed that at 4 and 24 weeks after the second booster, IgG levels were significantly higher in the COVID-19 group than in the non-COVID-19 group (Table 4; Fig. 3C through F).

## DISCUSSION

This study investigated the T and B cell responses following the first and second booster doses of BNT162b2 in healthcare workers who had previously received a two-dose regimen of CoronaVac. CD4+ and CD8+ T cell responses were detectable 4 weeks after the first booster; however, only CD4+ T cell responses persisted for at least 24 weeks after the second booster. These findings are consistent with previous studies demonstrating that CD4+ T cells are rapidly induced by vaccination and can be maintained for up to 6 months (29–33), whereas IgG antibody levels against the SARS-CoV-2-spike protein decline significantly within 3–4 months post-vaccination (34–37).

The substantially greater CD4+ and CD8+ T cell responses observed after the first booster dose of BNT162b2 compared to the second booster suggested a limited impact of the second booster on enhancing T cell immunity. Notably, CD8+ T cell responses were undetectable 4 weeks after the second booster, whereas CD4+ T cell responses were maintained, albeit at relatively low levels. These findings are consistent with those of a previous study indicating that mRNA vaccine boosters have minimal effects on CD8+ T cell activation (38).

During the 24-week follow-up period, nearly half of the participants developed COVID-19 at approximately 16 weeks after receiving the second booster dose of BNT162b2. Importantly, none of these individuals experienced severe disease, required hospitalization or ICU admission, or died. In this group, the significantly higher CD4+ T cell responses observed 24 weeks after the second booster compared to the non-COVID-19 group may reflect the contribution of natural infection-induced immunity. Conversely, the significantly higher CD4+ T cell responses at 4 weeks after the second booster in the non-COVID-19 group suggested that vaccine-induced T cell immunity may play a role in protection against SARS-CoV-2 infection and progression to severe disease.

Several studies have demonstrated the beneficial effects of both homologous and heterologous first booster doses of BNT162b2 on T cell responses (19–22, 39, 40). However, data on T cell responses in individuals who received a first BNT162b2 booster following a primary two-dose CoronaVac regimen remain limited (41, 42). These studies suggest that BNT162b2 enhances T cell-mediated immunity in CoronaVac-primed individuals. Nonetheless, evidence regarding the immunological effects of a second heterologous booster dose of BNT162b2 remains scarce. The present study suggests that administering a second heterologous booster dose of BNT162b2 following a primary two-dose regimen of CoronaVac induces early activation of both CD4+ and CD8+ T cell responses. Notably, CD4+ T cell responses were maintained at low but detectable levels up to 24 weeks post-vaccination, whereas CD8+ T cell responses declined rapidly and became undetectable by week 4 following the second booster. This pattern may reflect a limitation of the heterologous mRNA booster in generating long-lasting cytotoxic T cell-mediated immunity despite its activation potential.

IgG specific to the RBD of the SARS-CoV-2 spike protein has been identified as a key target of B cell-mediated immunity (43). In our study, memory B cells were detected at low frequencies at all three time points: 4 weeks after the first booster, and 4 and 24 weeks after the second BNT162b2 booster. This finding aligns with a previous report showing that memory B cells persist for up to 24 weeks after BNT162b2 vaccination (44).

A slight non-significant increase in the number of memory B cells was observed at 24 weeks compared to that at 4 weeks after the second booster. This may be attributed to the SARS-CoV-2 infections that occurred in some participants approximately 16 weeks after the second booster. To further investigate this possibility, we compared the memory B cell responses between the COVID-19 and non-COVID-19 groups, revealing that the number of memory B cells was comparable between the two groups at both time points. These findings suggest that SARS-CoV-2 infection following booster vaccination may have a limited effect on memory B cell levels. Although repeated vaccinations can contribute to the persistence of memory B cells, the overall effectiveness of protective immunity may still wane due to age-related immune senescence (45) or increased virulence of emerging SARS-CoV-2 variants.

Additionally, the persistence of memory B cells alone may not be sufficient to prevent infection by emerging SARS-CoV-2 variants. Neutralizing antibodies play a critical role in mediating protective immunity against viral infections. Individuals who experience a rapid decline in neutralizing antibody levels may remain susceptible to outbreaks. In the present study, IgG levels against the SARS-CoV-2 spike protein significantly declined at 24 weeks in the non-COVID-19 group. These findings support the hypothesis that levels of IgG antibodies wane over time. Our findings are also consistent with previous reports showing that IgG levels targeting the SARS-CoV-2-spike protein declined over time following the second BNT162b2 booster in individuals who had previously received a two-dose CoronaVac regimen (37). At 4 weeks after the second booster, IgG levels were significantly higher in the COVID-19 group than in the non-COVID-19 group. The occurrence of breakthrough infection despite high antibody titers suggests that elevated humoral immunity may not confer complete protection against SARS-CoV-2 infection, which may be attributed to several immunological and virological factors. First, antibody levels do not fully correlate with protection against infection. Instead, they reflect a strong serologic response but do not necessarily equate to sterilizing immunity. Second, antibody concentrations and neutralizing activity wane over time, leading to progressively reduced protection. Third, previous studies have demonstrated that emerging SARS-CoV-2 variants, particularly Omicron and its sublineages, exhibit significant immune escape (46–48). Omicron subvariants harbor multiple mutations within the spike protein, particularly in the RBD, thereby diminishing the binding affinity and neutralization efficiency of vaccine-induced antibodies. Finally, cell-mediated immunity, particularly memory CD4+ and CD8+ T cell responses, plays a more prominent role in limiting disease progression than in preventing infection. Consequently, individuals with robust T cell responses may still contract SARS-CoV-2, but typically experience milder clinical symptoms (49, 50).

This study had certain limitations. First, it was not a randomized controlled trial, and the sample size was relatively small, which may limit the generalizability of the findings. Second, the participants were not tested for SARS-CoV-2 infection at baseline; therefore, asymptomatic infections prior to enrollment could not be ruled out, potentially confounding the observed T and B cell responses. Third, T cell responses were assessed based on IFN-γ production as measured by ELISpot. However, this method does not provide information on other cytokines or the polyfunctionality of T cells. Therefore, the frequency of antigen-specific cells alone may not provide a comprehensive evaluation of T cell responses.

### Conclusions

The present study demonstrated that a heterologous second booster dose of BNT162b2, administered following a primary two-dose CoronaVac regimen for COVID-19 vaccination, rapidly induced CD4+ T and B cell responses that were maintained for at least 6 months.
